# Supplementary material for: Community pharmacy and general practice collaborative and integrated working: a realist review protocol
Source: BMJ Open. 2022 Dec 29;12(12):e067034. doi: 10.1136/bmjopen-2022-067034 (PMC9806063; doi:10.1136/bmjopen-2022-067034)
Supplement: Supplementary data [file bmjopen-2022-067034supp001.pdf]

Supplementary File 1

Details of the search developed for MEDLINE:

**Data Source**	Database	MEDLINE	
	Details	Medline (Ovid MEDLINE® Epub Ahead of Print, In-Process & Other Non-Indexed Citations, Ovid MEDLINE® Daily and Ovid MEDLINE®) 1946 to present	
	Host	Ovid	
	Date searched	18/04/2022	
	Database Update	Daily update	
**Limiters**	English only?	Yes	
	Time period searched	2000 to current	
	Publications types	No limit	
	Other		
**Results**	Items found	609	
	Internal duplicates (within one database)	0	
	External duplicates (between databases)	0	
	New	609	
In Ovid, save your search. Go to **Saved Searches** and click on the eye icon of your SR saved search to **View** your search. Copy and paste your search into **Notepad;** put tabs between the line number and text. Copy and paste into cell **B15**.		**Paste text of search strategy below**	
	**CompanionTest Version E MEDLINE**		
	Medline (Ovid MEDLINE® Epub Ahead of Print, In-Process & Other Non-Indexed Citations, Ovid MEDLINE® Daily and Ovid MEDLINE®) 1946 to present		
	1	((communit* or local) adj2 pharmac*).ti,ab,kw.	10064
	2	Community Pharmacy Services/	5409
	3	1 or 2	11999
	4	(general practi* or family practi* or family physician* or primary health* or primary care).ti,ab,kw.	249834
	5	exp *Primary Health Care/	106846
	6	exp *General Practice/	50899
	7	*general practitioners/ or *physicians, family/ or *physicians, primary care/	22059
	8	*Ambulatory Care/	20715
	9	or/4-8	348227
	10	((integrat* or collab* or cooperat* or co-operat* or coordinat* or co-ordinat* or interprofessional* or inter-professional* or multidisciplin* or multi-	158988
	11	((joint or joined-up or joined up or relations*) adj1 work*).ti,ab,kw.	4322
	12	((organi?ation* or delivery or care) adj1 model*).ti,ab,kw.	14362
	13	(infrastructure* or interface*).ti,ab,kw.	267499
	14	*Attitude of Health Personnel/	65859
	15	*Cooperative Behavior/	18726
	16	exp *Interprofessional Relations/	29750
	17	exp *Professional Role/	46346
	18	exp *"Delivery of Health Care, Integrated"/	10524
	19	*models, organizational/	6488
	20	or/10-19	583426
	21	9 and 20	43231
	22	Primary Health Care/og [Organization & Administration]	15886
	23	21 or 22	54656
	24	3 and 23	669
	25	limit 24 to (english language and yr="2000 -Current")	609
