# Supplementary material for: Community pharmacy and general practice collaborative and integrated working: a realist review protocol
Source: BMJ Open. 2022 Dec 29;12(12):e067034. doi: 10.1136/bmjopen-2022-067034 (PMC9806063; doi:10.1136/bmjopen-2022-067034)
Supplement: Author's manuscript [file bmjopen-2022-067034.draft_revisions.pdf]

BMJ Open

BMJ Open is committed to open peer review. As part of this commitment we make the peer review history of every article we publish publicly available.

When an article is published we post the peer reviewers' comments and the authors' responses online. We also post the versions of the paper that were used during peer review. These are the versions that the peer review comments apply to.

The versions of the paper that follow are the versions that were submitted during the peer review process. They are not the versions of record or the final published versions. They should not be cited or distributed as the published version of this manuscript.

BMJ Open is an open access journal and the full, final, typeset and author-corrected version of record of the manuscript is available on our site with no access controls, subscription charges or pay-per-view fees (http://bmjopen.bmj.com).

If you have any questions on BMJ Open's open peer review process please email info.bmjopen@bmj.com

BMJ Open

Community Pharmacy and General Practice collaborative and integrated working: a realist review protocol

Journal:	*BMJ Open*
Manuscript ID	bmjopen-2022-067034
Article Type:	Protocol
Date Submitted by the Author:	05-Aug-2022

Complete List of Authors:	Owen, Emily; University College London, Department of Primary Care & Population Health<br>Whittlesea, Cate; University College London, Research Department of Practice and Policy, UCL School of Pharmacy<br>Duddy, Claire; University of Oxford, Nuffield Department of Primary Care Health Sciences<br>Swinglehurst, Deborah; Barts and The London School of Medicine and Dentistry, Institute of Population Health Sciences;<br>Husson, Fran; University College London, Department of Primary Care & Population Health ; Imperial College London, Faculty of Medicine<br>Wong, Geoff; Oxford University, Nuffield Department of Primary Care Health Sciences<br>Hamer-Hunt, Julia; University College London, Department of Primary Care & Population Health; University of Oxford, Dept of Psychiatry<br>Mahtani, Kamal; University of Oxford, Nuffield Department of Primary Health Care Sciences<br>Turner, Malcolm ; University College London, Department of Primary Care and Population Health<br>Ogden , Margaret; University College London, Department of Primary Care and Population Health<br>Fudge, Nina ; Queen Mary University of London, Wolfson Institute of Population Health Sciences<br>Abrams, Ruth; University of Surrey, School of Health Sciences, Faculty of Health and Medical Sciences<br>Cai, Ziyue ; University College London, Research Department of Practice and Policy, UCL School of Pharmacy<br>Park, Sophie; University College London, Department of Primary Care and Population Health

Keywords:	Organisation of health services < HEALTH SERVICES ADMINISTRATION & MANAGEMENT, Quality in health care < HEALTH SERVICES ADMINISTRATION & MANAGEMENT, Public health < INFECTIOUS DISEASES, PRIMARY CARE

SCHOLARONE™
Manuscripts

RUNNING HEAD: REALIST REVIEW PROTOCOL

Community Pharmacy and General Practice collaborative and integrated working: a realist review protocol

Emily C. Owen[1], Cate Whittlesea[2], Claire Duddy[3], Deborah Swinglehurst[4], Fran Husson[1,5], Geoff Wong[3], Julia Hamer-Hunt[1,6], Kamal R. Mahtani[3], Malcolm Turner[1], Margaret Ogden[1], Nina Fudge[4], Ruth Abrams[7], Ziyue Cai[2], Sophie Park[1]

[1]Department of Primary Care and Population Health, University College London, London, UK, [2]Research Department of Practice and Policy, School of Pharmacy, University College London, London, UK, [3]Nuffield Department of Primary Care Health Sciences, University of Oxford, Oxford, UK, [4]Wolfson Institute of Population Health Sciences, Barts and The London School of Medicine and Dentistry, Queen Mary University of London, London, UK, [5]Faculty of Medicine, Imperial College London, London, UK, [6]Department of Psychiatry, University of Oxford, Oxford, UK, [7]School of Health Sciences, University of Surrey, Surrey, UK.

Corresponding author:

Dr Emily C. Owen; UCL Research Department of Primary Care & Population Health; Royal Free Campus; Hampstead, London; NW3 2PY.

Email: Emily.owen@ucl.ac.uk; Phone: 020 7794 0500.

Keywords: Community Pharmacy; General Practice; Primary Health Care; Delivery of Health Care; Public Health.

Word count: 3062.

For peer review only

ABSTRACT

Introduction Increasing collaborative and integrated working between General Practice (GP) and Community Pharmacy (CP) is a key priority of the UK National Health Service (NHS) and has been proposed as a solution to reducing health system fragmentation and improving synergies and coordination of care. However, there is limited understanding regarding how and under which circumstances collaborative and integrated working between GP and CP can be achieved in practice and how regulatory, organisational, and systemic barriers can be overcome.

Methods and analysis The aim of our review is to understand how, when, and why working arrangements between GP and CP can provide the conditions necessary for optimal communication, decision-making, and collaborative and integrated working. A realist review approach will be used to synthesise the evidence to make sense of the complexities inherent in the working relationships between GP and CP. Our review will follow Pawson's five iterative stages: (1) finding existing theories; (2) searching for evidence; (3) article selection; (4) data extraction; and (5) synthesising evidence and drawing conclusions. We will synthesise evidence from grey literature, qualitative, quantitative, and mixed-methods research. The research team will work closely with key stakeholders and public contributor co-applicants (PPI) throughout the review process to refine the focus of the review and the programme theory. Collectively, our refined programme theory will explain how collaborative and integrated working between GP and CP works (or not), for whom, how, and under which circumstances.

Ethics and dissemination Formal ethical approval is not required for this review as it draws on secondary data from published articles and grey literature. Findings will be widely

For peer review only

disseminated through: publication in peer-reviewed journals; seminars; international conference presentations; patients' association channels, social media, symposia; and user-friendly summaries.

PROSPERO registration number: CRD42022314280.

Strengths and limitations of this study

- This is the first realist review to explore collaborative and integrated working between general practice and community pharmacy. Consequently, this review responds to the need to understand the contexts and mechanisms through which collaboration and integration may (or may not) lead to effective and equitable healthcare outcomes.
- Public contributor co-applicants and key stakeholders will contribute to the development and refinement of the programme theory, analysis, interpretation, dissemination of findings, and help to ensure our research informs future policy and practice.
- The review findings may be transferable to other primary care interfaces and the future productive shaping of integrated and collaborative working.
- Our review may be limited by the quality and relevance of existing literature in this field.
- We will only include literature that is written or translated into English.

For peer review only

INTRODUCTION

The NHS Long-Term Plan (1) sets out fundamental changes to the nature and provision of UK primary care. This includes the introduction of ‘Integrated Care Systems’ (ICSs) and legislative changes to support collaborative and integrated working between individuals and institutional organisations. It recommends significant expansion in the numbers of allied healthcare professionals working in primary care settings, and related extension in their roles and responsibilities. For instance, the role of CPs has evolved from *supporting* GP (e.g., Medicines Use Review) towards *joint working* (2).

There are many different definitions used in the literature to describe collaborative and integrated working between healthcare professionals. Some definitions indicate that collaboration involves shared goals, decision-making, trust, and open, honest communication (3). Integration may include common policies and incentives, defined referral mechanisms, practice guidelines, and formal structures (3, 4). Increased collaborative and integrated working between GP and CP may have potential to enhance access to services and improve interprofessional communication and continuity of patient care (5). Policy direction and expectations are clear: shifting away from competition towards more collaborative ways of working (6). Examples include recent National Institute for Health and Care Excellence (NICE) standards to facilitate effective partnership working (7), ‘flu vaccine principles (8), and enhanced service amendments (9) for GP referrals to CP. However, implementing these changes has created unanticipated consequences. For instance, the more clinical activity is distributed across providers, healthcare disciplines, and settings, the greater the risk for silo working, fragmentation of accountability and patient care (10). To enable effective collaborative and integrated working, there remains a need to develop an in-depth understanding about how these providers work together; what influences interactions; and how these impact patient trust, experience, and outcomes.

For peer review only

Policy changes have the potential to influence how patients' access, use, and experience primary care; form and sustain relationships with healthcare professionals; and navigate healthcare expertise within and between clinical encounters. This has implications for the nature of work done by professional groups including how this work is achieved, the use of technology to support working, how accountability (clinical and financial) is determined, the training and capacity building required; and importantly, how healthcare professionals work with each other to support effective, safe, and equitable patient care.

UK reports in the last decade consistently position CP as a solution for over-burdened GP and a means to improve patient access, providing 'health on the high street' (11, 12), including Directed Enhanced Services (13). This has been part of the rationale behind the Long-Term Plan's goal of reorganising primary care so that CP is included in wider networks such as Primary Care Networks (PCNs) and ICSs (1). However, implementation of collaborative working amongst independent contractors to the NHS, such as GP and CP, can be complex and problematic.

Professional bodies, including the Royal Pharmaceutical Society (14), have published recommendations to help CP integrate into primary care (15). However, these are not specific to ICSs and PCNs, and predominantly focus on tighter technology integration to facilitate communication between GP and CP. Recent guidance (8) includes expectations for integrated services, referral pathways, and joint strategic needs assessments to address local health inequalities, but does not describe how these should be achieved.

Previous reviews have demonstrated that CP could deliver effective patient care in several areas, but implementation depends on successful collaboration and integration with other services (5, 16). Existing research into collaborative working in these settings found that imbalances of authority, limited understanding of others' roles and responsibilities, lack of time and financial remuneration, and professional boundary friction when delivering

For peer review only

patient care, can impact on the quality of care provided (5, 17, 18). Collaboration and integrated working may bring new roles and ways of working for healthcare professionals in each organisation. These changes may also, however, contribute to and exacerbate ambiguity about professional roles and boundaries, potentially undermining the collaborative and integrated working between GP and CP, with resulting confusion for patients (19). There is, therefore, a potential paradox at play, in which efforts to create greater role clarity, professional boundaries, and distribution of work tasks in the service of integrating care contribute to poorly integrated patient care. Extant literature is not, however, able to explain when (in what contexts), why, and how the identified factors affect working between GP and CP. Whilst current evidence indicates that encouraging GP and CP to work together is important, it is less clear how these working arrangements can be made to work well within ICS settings for both healthcare professionals and patients.

METHODS

Review aim, questions, and objectives

Aim

This review aims to understand how, when, and why working arrangements between GP and CP can provide the conditions necessary for optimal communication, decision-making, and collaborative and integrated working.

Research Objectives

1. Develop a programme theory through an evidence synthesis of how GP and CP can optimise communication, decision-making, and collaborative and integrated working to support effective and equitable patient care.

For peer review only

2. Embed and respond to stakeholder and PPI perspectives throughout the design, analysis, and report stages of the project, thus maximising the relevance and utility of review findings.
3. Make recommendations for practice and policy based on the refined programme theory.

Research Questions:

Within the existing literature, what can we learn that will help GP and CP to work together in a collaborative and integrated way to support effective and equitable healthcare outcomes? Specifically, the review will be guided by the following questions:

1. What are the mechanisms which support GP and CP to work in an integrated and collaborative way?
2. What are the important contexts which influence whether different mechanisms produce intended and unintended outcomes in GP and CP working relationships?
3. What are the interventional strategies that are likely to lead to intended and unintended outcomes within GP and CP working relationships?

Approach

A realist review is an interpretive and theory-driven approach to synthesising evidence from grey literature (e.g., policy documentation), qualitative, quantitative, and mixed-methods research (20). It enables the use of a range of data types to make sense of and address the context-sensitive outcomes arising from interactions between GP and CP. The realist approach begins by developing an initial programme theory that aims to explain how collaborative and integrated working between GP and CP may (or may not) work. This initial programme theory is then tested (confirmed, refuted, or refined) against empirical evidence

For peer review only

throughout the review. The final phase of a realist review involves the synthesis of evidence, the formulation of context-mechanism-outcome configurations (CMOCs), and a refined programme theory which explains whether, why, how, and to what extent GP and CP collaborative and integrated working practices may support effective and equitable healthcare delivery for patients (21). A realist review has the potential to produce meaningful, transferable findings across different structures and contexts within which GP and CP operate.

Patient and Public Involvement (PPI)

We will consult with stakeholders with a wide range of relevant expertise comprising: four PPI co-applicants (including family and carer perspectives); front-line healthcare clinicians; workforce and training experts; and a multi-disciplinary team of researchers. The project team (including PPI co-applicants) will meet every two months, working collaboratively alongside experiential stakeholders. There will be three stakeholder meetings (lasting approximately 3-hours), including representation from small, medium, and large pharmacy and medical organisations. Throughout the project, detailed notes will be made during and after each meeting, and the outcomes of our engagement with PPI co-applicants and stakeholders will be recorded using an impact log (22). Collectively, our PPI co-applicants and stakeholder groups will contribute to the development and refinement of the programme theory, analysis, interpretation, dissemination activities (e.g., co-presenting and co-publishing papers, posters, presentations, and reports), and help to ensure our research informs future practice and policy in this important area of inquiry. This realist review protocol is registered in PROSPERO (reference number: CRD42022314280) and will be carried out in accordance with the quality and publication standards (23, 24).

Step 1: Finding Existing Theories

For peer review only

We will identify theories that explain how collaborative and integrated working between GP and CP is expected to 'work' to generate effective and equitable healthcare outcomes (23). To do this, we will consult with key content experts in our stakeholder group and informally scope existing literature identified via citation tracking and snowballing (25), which will also include identifying any formal theories that may be of relevance (26). We will use relevant theories and data we find in this step to develop our initial programme theory, which will be further tested and scrutinised in this review. A draft of our initial programme theory is provided in Figure 1.

Figure 1 Initial programme theory – used at the beginning of realist review projects to map initial explanatory theories.

****Insert Figure 1 Around Here****

Step 2: Searching for Evidence

We will conduct systematic searches to identify a relevant 'body of literature' with which to develop and refine the programme theory from step 1. The main search strategy was designed, piloted, and conducted by CD in collaboration with ECO and the project team. Our searches combined free text and subject heading (e.g., MeSH) terms for GP and CP with a range of terms describing collaborative and integrated working arrangements. The full details of the search developed for MEDLINE are available in Supplementary File 1. This search strategy was translated for use in the following databases: MEDLINE (Ovid); Embase (Ovid); CINAHL (Ebsco); PsycINFO (Ovid); HMIC (Ovid); International Bibliography of the Social Sciences (ProQuest); Sociology Collection (ProQuest); the Web of Science (SSIE, SSCI, ESCI, and CPCI indexes); and the King's Fund Library database. This search identified 2555 potentially relevant documents, of which 1133 were duplicates. A total of 1422 unique references were identified for screening.

For peer review only

At later stages of this review, we may undertake additional searching, including searches of any other relevant databases identified by CD, 'cited by' article searches, and searches of the citations contained in the reference lists of relevant documents. Additional grey literature e.g., documents produced by the Department of Health, local Clinical Commissioning Groups (or successor organisations), and pharmacists' professional groups will also be identified via searches of relevant websites. Relevant evidence including opinion and commentary will be used to inform programme theory development, along with published and unpublished evaluations of existing integrated services and collaborations which are likely to provide more detailed local contextual information.

Based on our preliminary searches, our initial inclusion criteria will include GP and CP; UK (the initial focus will be on the UK and countries with a universal healthcare system but we may draw on data from other healthcare systems); Date 2000 onwards (in order to capture literature prior to the first integrated and collaborative initiative called Medicines Use Review in 2003); and a focus on an element of the 'working relationship' between GP and CP (to include terms such as integrated and collaborative working, but not exclusively). We expect to exclude papers focused entirely on clinical pharmacists working within GP settings due to differences in roles, responsibilities, and the professional framework of community and clinical pharmacists.

Step 3: Article Selection

This is a three-stage process: screening against title/abstract; then by full text; and finally full text documents will be selected based on their relevance (whether they contain data to contribute to theory building and/or testing) and rigour (whether the methods used to generate the relevant data are credible and trustworthy) (20). To ensure consistency, a random 10%

For peer review only

sample of decisions will be independently checked at each stage by CD and/or NF. Any discrepancies or disagreements will be discussed with the research team and documented.

Step 4: Data Extraction

Data extraction and organisation will be undertaken by ECO. Discrepancies or disagreements will be discussed in detail and documented. The included full texts will be uploaded into qualitative data analysis software (EPPI-Reviewer Web) for coding. These will be both coded deductively (codes created in advance of data extraction and analysis, as informed by the initial programme theory), inductively (codes created to categorise data reported in included studies), and retroductively (codes created based on an interpretation of data to infer what the hidden causal forces might be for outcomes). Each new element of relevant data will be used to refine the programme theory, and as it is refined, included studies will be re-scrutinised to search for relevant data that may have been missed initially. A random sample of 10% extracted data and coding will be independently checked by CD or NF for quality control.

Step 5: Synthesising the Evidence and Drawing Conclusions

Data analysis will use a realist logic of analysis to make sense of the initial programme theory. ECO will undertake this step with support from the research team, PPI, and stakeholders. We will use interpretive cross-case comparison to understand and explain how and why observed outcomes have occurred, for example, by comparing literature in which GP and CP have successfully worked collaboratively against those which have reported the interface as unsuccessful or detrimental, to understand how context has influenced reported findings.

For peer review only

We will use an established analysis and synthesis process (26). In brief, to operationalise the realist logic of analysis, we will ask the following questions:

- Interpretation of meaning: do the documents provide data that may be interpreted as functioning as context, mechanism, or outcome?
- Interpretations and judgements about CMOCs: what is the CMOC for the data that has been interpreted as functioning as context, mechanism, or outcome?
- Interpretations and judgements about programme theory: how does this CMOC relate to the initial programme theory?

Ethics and Dissemination

Dissemination

Our PPI contributors and stakeholders will help us to decide on the content, storyboarding, and format (e.g., websites, leaflets, videos, social media). In addition to the final report, we will produce outputs directed at diverse audiences:

1. **Academic outputs**: e.g., protocol publication. Findings (to be submitted to a high-impact, open-access, and peer-reviewed journal), as well as tailored papers to different disciplinary journals.
2. **Audience-specific practitioner** 'how to' publications which outline practice advice on how to optimise GP and CP collaborative and integrated working for patient benefit.
3. **User-friendly summaries** of the review findings tailored to the needs of different audiences including the public and service users.

Our dissemination strategy will build upon a participatory approach, embedding PPI and stakeholder involvement throughout the development of this research and project timeline, including opportunities for co-authorship and co-presenting. On-going engagement with key

For peer review only

stakeholders will maximise opportunities to utilise our established networks, communication channels, and links to policymakers and providers. Our approach will be integrative, valuing the different forms of knowledge needed to produce findings capable of informing complex decision-making (27).

Ethics

Ethical approval is not required for this review as only secondary data sources will be used.

DISCUSSION

Importance of the Research

This review will provide insights and solutions to maximise GP and CP collaboration and integration. The findings and refined programme theories will ensure patients' health and healthcare experience is central to GP and CP working relationships and processes. These working relationships and arrangements impact on patient experience, patient safety and medication errors, access, care, and formal referral, alongside professional capacity, training, and workload. The review findings are likely to have broader relevance to other primary care interfaces and the future productive shaping of integrated and collaborative working.

Authors' contributions

The realist review was conceptualised by SP. ECO wrote the first draft of this protocol and it was reviewed and revised by SP, CW, CD, DS, FH, GW, JHH, KRM, MT, MO, NF, RA, ZC. CD designed, piloted, and conducted the search strategy in collaboration with ECO and the project team. All authors have read and approved the final manuscript.

Funding Acknowledgement

For peer review only

This project is funded by the National Institute for Health Research (NIHR) School for Primary Care Research (project reference 567988). The views expressed are those of the authors and not necessarily those of the NIHR or the Department of Health and Social Care.

Competing interests

The authors have no conflicts of interests to declare.

For peer review only

References

1. NHS E. The NHS Long Term Plan 2019 [Available from: https://www.longtermplan.nhs.uk/wp-content/uploads/2019/08/nhs-long-term-plan-version-1.2.pdf.

2. Health Do. A vision for pharmacy in the new NHS 2003 [Available from: http://data.parliament.uk/DepositedPapers/Files/DEP2009-0485/DEP2009-0485.pdf.

3. Morley L, Cashell A. Collaboration in Health Care. J Med Imaging Radiat Sci. 2017;48(2):207-16.

4. Boon HS, Mior, S. A, Barnsley, J, Ashbury, F. D, Haig, R. The difference between integration and collaboration in patient care: results from key informant interviews working in multiprofessional health care teams. J Manipulative Physiol Ther. 2009;32(9):715-22.

5. Bollen A, Harrison R, Aslani P, van Haastregt JCM. Factors influencing interprofessional collaboration between community pharmacists and general practitioners-A systematic review. Health Soc Care Community. 2019;27(4):e189-e212.

6. NHS E. We are the NHS: people plan 2020-21—action for us all. 2020 [Available from: https://www.england.nhs.uk/publication/we-are-the-nhs-people-plan-for-2020-21-action-for-us-all/.

7. (NICE) NIfHaCE. Community pharmacies: promoting health and wellbeing 2020 [Available from: https://www.nice.org.uk/guidance/qs196/resources/community-pharmacies-promoting-health-and-wellbeing-pdf-75545785198789.

8. Practitioners RPSaRCoG. Influenza vaccination: Principles for collaboration across Great Britain 2020 [Available from: https://www.rpharms.com/Portals/0/RPS%20document%20library/Open%20access/Flu%20vaccinations/Flu%20Vaccination%20Joint%20Statement.pdf.

9. ACT TNHS. The Pharmaceutical Services (Advanced and Enhanced Services and Emergency Declaration) (England) (Amendment) (No. 2) Directions 2020 2006 [Available from: https://www.nhsbsa.nhs.uk/sites/default/files/2020-08/Pharmaceutical%20Services%20%28Adv%20Enh%20%20Emerg%20Declar%29%20%28Eng%29%20%28Flu%20vacc%20Hep%20C%20etc%29%20Dir%2028082020.pdf.

10. Sevin C, Moore G, Shepherd J, Jacobs T, Hupke C. Transforming care teams to provide the best possible patient-centered, collaborative care. J Ambul Care Manage. 2009;32(1):24-31.

11. Alliance N. Supporting the Development of Community Pharmacy Practice within Primary Care 2016 [Available from: https://nhsalliance.org/wp-content/uploads/2018/11/Supporting-the-Development-of-Community-Pharmacy-Practice-within-Primary-Care-NEW-LOGO.pdf.

12. Smith J, Picton, C., Dayan, M NOW OR NEVER: shaping pharmacy for the future. 2013

13. Committee DPSN. A summary of the Network Contract Directed Enhanced Service (DES) 2019/20 2019 [Available from: https://psnc.org.uk/wp-content/uploads/2019/04/PSNC-Briefing-018.19-A-summary-of-the-Network-Contract-Directed-Enhanced-Service-DES-2019.20.pdf.

14. Society. RP. The future of pharmacy in a sustainable NHS: Key principles for transformation and growth 2022 [Available from: https://www.rpharms.com/recognition/all-our-campaigns/policy-a-z/future-of-pharmacy.

15. Body PRS. Community Pharmacy Standard Final Report 2022.

16. Milosavljevic A, Aspden T, Harrison J. Community pharmacist-led interventions and their impact on patients' medication adherence and other health outcomes: a systematic review. Int J Pharm Pract. 2018;26(5):387-97.

For peer review only

17. Hossain LN, Fernandez-Llimos F, Luckett T, Moullin JC, Durks D, Franco-Trigo L, et al. Qualitative meta-synthesis of barriers and facilitators that influence the implementation of community pharmacy services: perspectives of patients, nurses and general medical practitioners. BMJ Open. 2017;7(9):e015471.

18. Hindi AMK, Schafheutle EI, Jacobs S. Community pharmacy integration within the primary care pathway for people with long-term conditions: a focus group study of patients', pharmacists' and GPs' experiences and expectations. BMC Fam Pract. 2019;20(1):26.

19. Karampatakis GD, Patel N, Stretch G, Ryan K. Community pharmacy teams' experiences of general practice-based pharmacists: an exploratory qualitative study. BMC Health Serv Res. 2020;20(1):431.

20. Pawson R. Evidence-Based Policy: A Realist Perspective London: SAGE Publications.; 2006.

21. Pawson R, & Tilley, N Realistic Evaluation SAGE Publications; 1997.

22. Kok M. Guidance Document: Evaluating public involvement in research 2018 [Available from: http://www.phwe.org.uk/wp-content/uploads/Guidance-on-evaluating-Public-Involvement-in-research.pdf.

23. Wong G, Greenhalgh T, Westhorp G, Buckingham J, Pawson R. RAMESES publication standards: realist syntheses. BMC Med. 2013;11:21.

24. Wong G, Greenhalgh T, Westhorp G, Pawson R. Development of methodological guidance, publication standards and training materials for realist and meta-narrative reviews: the RAMESES (Realist And Meta-narrative Evidence Syntheses - Evolving Standards) project. Health Services and Delivery Research. Southampton (UK)2014.

25. Greenhalgh T, Peacock R. Effectiveness and efficiency of search methods in systematic reviews of complex evidence: audit of primary sources. BMJ. 2005;331(7524):1064-5.

26. Booth A, Harris J, Croot E, Springett J, Campbell F, Wilkins E. Towards a methodology for cluster searching to provide conceptual and contextual "richness" for systematic reviews of complex interventions: case study (CLUSTER). BMC Med Res Methodol. 2013;13:118.

27. Bowen SG, I Integrated knowledge translation. In: Straus S, & Tetroe, J editor. Knowledge translation in health care: Moving from evidence to practice. Oxford: Wiley Blackwell 2013 p. 14-23.

For peer review only

Possible wider influences on effective communications:

- Provider contractual requirements (e.g., independent contractor status to the NHS)
- Funding models for General Practices and Pharmacies
- Rules, regulations, and policies
- Referral processes
- NHS England policy drivers (e.g., Long Term Plan to working collaboratively at scale)
- Professional identities
- Organisational cultures within General Practices and Pharmacies

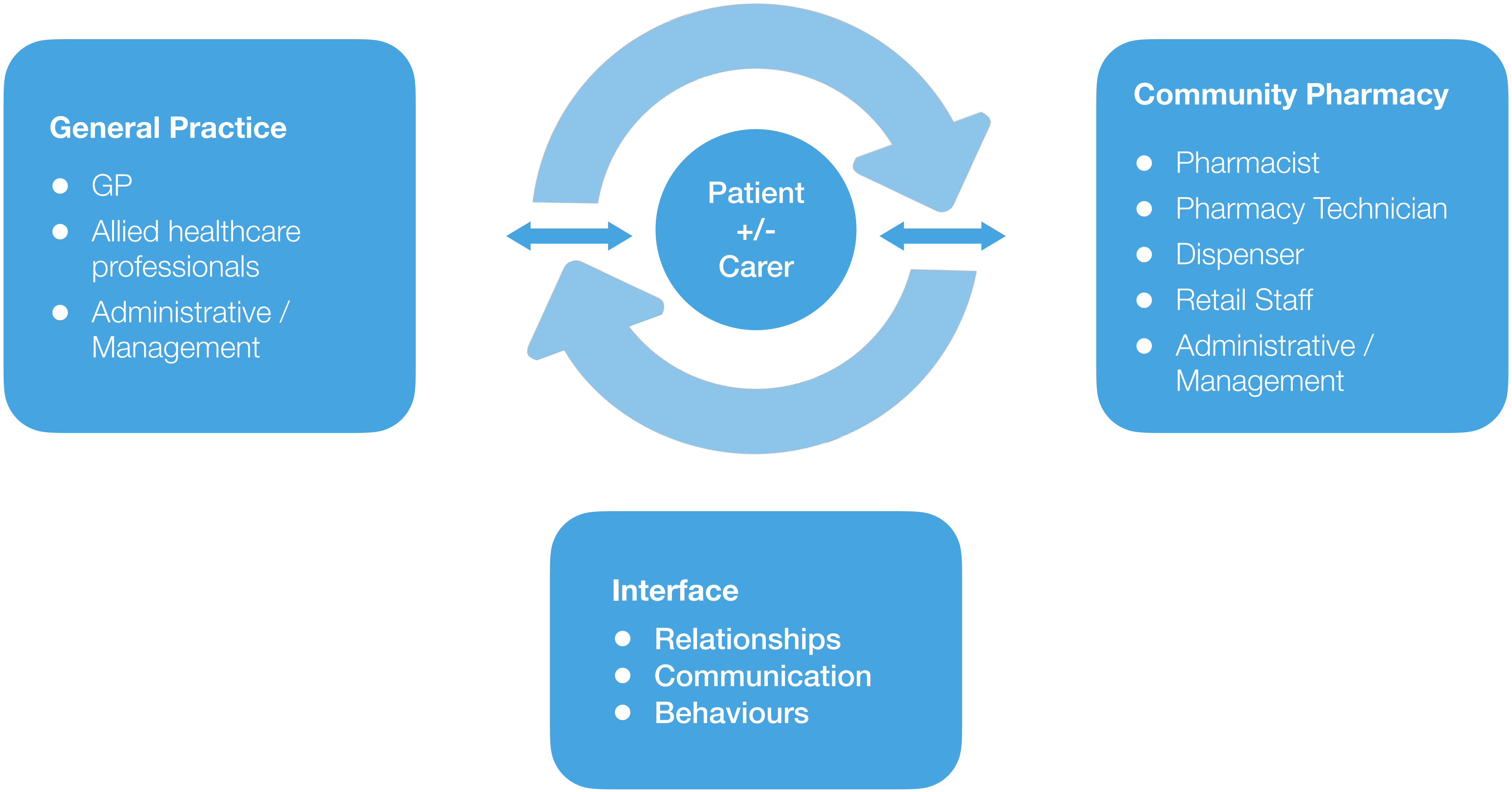

Supplementary File 1

Details of the search developed for MEDLINE:

**Data Source**	Database	MEDLINE	
	Details	Medline (Ovid MEDLINE® Epub Ahead of Print, In-Process & Other Non-Indexed Citations, Ovid MEDLINE® Daily and Ovid MEDLINE®) 1946 to present	
	Host	Ovid	
	Date searched	18/04/2022	
	Database Update	Daily update	
**Limiters**	English only?	Yes	
	Time period searched	2000 to current	
	Publications types	No limit	
	Other		
**Results**	Items found	609	
	Internal duplicates (within one database)	0	
	External duplicates (between databases)	0	
	New	609	
In Ovid, save your search. Go to **Saved Searches** and click on the eye icon of your SR saved search to **View** your search. Copy and paste your search into **Notepad;** put tabs between the line number and text. Copy and paste into cell **B15**.	**Paste text of search strategy below**		
	**CompanionTest Version E MEDLINE**		
	Medline (Ovid MEDLINE® Epub Ahead of Print, In-Process & Other Non-Indexed Citations, Ovid MEDLINE® Daily and Ovid MEDLINE®) 1946 to present		
	1	((communit* or local) adj2 pharmac*).ti,ab,kw.	10064
	2	Community Pharmacy Services/	5409
	3	1 or 2	11999
	4	(general practi* or family practi* or family physician* or primary health* or primary care).ti,ab,kw.	249834
	5	exp *Primary Health Care/	106846
	6	exp *General Practice/	50899
	7	*general practitioners/ or *physicians, family/ or *physicians, primary care/	22059
	8	*Ambulatory Care/	20715
	9	or/4-8	348227
	10	((integrat* or collab* or cooperat* or co-operat* or coordinat* or co-ordinat* or interprofessional* or inter-professional* or multidisciplin* or multi-	158988
	11	((joint or joined-up or joined up or relations*) adj1 work*).ti,ab,kw.	4322
	12	((organi?ation* or delivery or care) adj1 model*).ti,ab,kw.	14362
	13	(infrastructure* or interface*).ti,ab,kw.	267499
	14	*Attitude of Health Personnel/	65859
	15	*Cooperative Behavior/	18726
	16	exp *Interprofessional Relations/	29750
	17	exp *Professional Role/	46346
	18	exp *"Delivery of Health Care, Integrated"/	10524
	19	*models, organizational/	6488
	20	or/10-19	583426
	21	9 and 20	43231
	22	Primary Health Care/og [Organization & Administration]	15886
	23	21 or 22	54656
	24	3 and 23	669
	25	limit 24 to (english language and yr="2000 -Current")	609

For peer review only

For peer review only

BMJ Open

Community Pharmacy and General Practice collaborative and integrated working: a realist review protocol

Journal:	*BMJ Open*
Manuscript ID	bmjopen-2022-067034.R1
Article Type:	Protocol
Date Submitted by the Author:	22-Nov-2022
Complete List of Authors:	Owen, Emily; University College London, Department of Primary Care & Population Health<br>Whittlesea, Cate; University College London, Research Department of Practice and Policy, UCL School of Pharmacy<br>Duddy, Claire; University of Oxford, Nuffield Department of Primary Care Health Sciences<br>Swinglehurst, Deborah; Barts and The London School of Medicine and Dentistry, Institute of Population Health Sciences;<br>Husson, Fran; University College London, Department of Primary Care & Population Health ; Imperial College London, Faculty of Medicine<br>Wong, Geoff; Oxford University, Nuffield Department of Primary Care Health Sciences<br>Hamer-Hunt, Julia; University College London, Department of Primary Care & Population Health; University of Oxford, Dept of Psychiatry<br>Mahtani, Kamal; University of Oxford, Nuffield Department of Primary Health Care Sciences<br>Turner, Malcolm ; University College London, Department of Primary Care and Population Health<br>Ogden , Margaret; University College London, Department of Primary Care and Population Health<br>Fudge, Nina ; Queen Mary University of London, Wolfson Institute of Population Health Sciences<br>Abrams, Ruth; University of Surrey, School of Health Sciences, Faculty of Health and Medical Sciences<br>Cai, Ziyue ; University College London, Research Department of Practice and Policy, UCL School of Pharmacy<br>Park, Sophie; University College London, Department of Primary Care and Population Health
**Primary Subject Heading**:	Public health
Secondary Subject Heading:	Health services research, General practice / Family practice, Communication
Keywords:	Organisation of health services < HEALTH SERVICES ADMINISTRATION & MANAGEMENT, Quality in health care < HEALTH SERVICES ADMINISTRATION & MANAGEMENT, Public health < INFECTIOUS DISEASES, PRIMARY CARE

RUNNING HEAD: REALIST REVIEW PROTOCOL

Community Pharmacy and General Practice collaborative and integrated working: a realist review protocol

Emily C. Owen[1], Ruth Abrams[2], Ziyue Cai[3], Claire Duddy[4], Nina Fudge[5], Julia Hamer-Hunt[1,6], Fran Husson[1,7], Kamal R. Mahtani[4], Margaret Ogden[1], Deborah Swinglehurst[5], Malcolm Turner[1], Cate Whittlesea[3], Geoff Wong[4], Sophie Park[1]

[1]Department of Primary Care and Population Health, University College London, London, UK, [2]School of Health Sciences, University of Surrey, Surrey, UK, [3]Research Department of Practice and Policy, School of Pharmacy, University College London, London, UK, [4]Nuffield Department of Primary Care Health Sciences, University of Oxford, Oxford, UK, [5]Wolfson Institute of Population Health Sciences, Barts and The London School of Medicine and Dentistry, Queen Mary University of London, London, UK, [6]Department of Psychiatry, University of Oxford, Oxford, UK, [7]Faculty of Medicine, Imperial College London, London, UK.

Corresponding author:

Dr Emily C. Owen; UCL Research Department of Primary Care & Population Health; Royal Free Campus; Hampstead, London; NW3 2PY.

Email: Emily.owen@ucl.ac.uk; Phone: 020 7794 0500.

Keywords: Community Pharmacy; General Practice; Primary Health Care; Delivery of Health Care; Public Health.

Word count: 3135.

For peer review only

ABSTRACT

Introduction Increasing collaborative and integrated working between General Practice (GP) and Community Pharmacy (CP) is a key priority of the UK National Health Service (NHS) and has been proposed as a solution to reducing health system fragmentation, improving synergies, and coordination of care. However, there is limited understanding regarding how and under which circumstances collaborative and integrated working between GP and CP can be achieved in practice and how regulatory, organisational, and systemic barriers can be overcome.

Methods and analysis The aim of our review is to understand how, when, and why working arrangements between GP and CP can provide the conditions necessary for optimal communication, decision-making, and collaborative and integrated working. A realist review approach will be used to synthesise the evidence to make sense of the complexities inherent in the working relationships between GP and CP. Our review will follow Pawson's five iterative stages: (1) finding existing theories; (2) searching for evidence (our main searches were conducted in April 2022); (3) article selection; (4) data extraction; and (5) synthesising evidence and drawing conclusions. We will synthesise evidence from grey literature, qualitative, quantitative, and mixed-methods research. The research team will work closely with key stakeholders and include Patient and Public Involvement and Engagement (PPIE) throughout the review process to refine the focus of the review and the programme theory. Collectively, our refined programme theory will explain how collaborative and integrated working between GP and CP works (or not), for whom, how, and under which circumstances.

For peer review only

Ethics and dissemination Formal ethical approval is not required for this review as it draws on secondary data from published articles and grey literature. Findings will be widely disseminated through: publication in peer-reviewed journals; seminars; international conference presentations; patients' association channels; social media; symposia; and user-friendly summaries.

PROSPERO registration number: CRD42022314280.

Strengths and limitations of this study

- This is the first realist review to explore collaborative and integrated working between general practice and community pharmacy.
- Public contributor co-applicants and key stakeholders will contribute to the development and refinement of the programme theory, analysis, interpretation, dissemination of findings, and help to ensure our research informs future practice.
- The review findings may be transferable to other primary care interfaces and the future productive shaping of integrated and collaborative working.
- Our review may be limited by the quality and relevance of existing literature in this field.
- We will only include literature that is written or translated into English.

For peer review only

INTRODUCTION

The NHS Long-Term Plan (1) sets out fundamental changes to the nature and provision of UK primary care. This includes the introduction of 'Integrated Care Systems' (ICSs) and legislative changes to support collaborative and integrated working between individuals and institutional organisations. It recommends significant expansion in the numbers of allied healthcare professionals working in primary care settings, and related extension in their roles and responsibilities. For instance, the role of Community Pharmacy (CP) has evolved from *supporting* General Practice (GP) (e.g., Medicines Use Review) towards *joint working* (2, 3).

There are many different definitions used in the healthcare literature to describe collaborative and integrated working between health professionals. Some definitions indicate that collaboration involves shared goals and responsibility, decision-making, trust, and open, honest communication (4). Integration may include common policies and incentives, defined referral mechanisms, practice guidelines, and formal structures (4, 5). Effective collaborative and integrated working between GP and CP may have the potential to enhance access to services, improve interprofessional communication, and continuity of patient care (6). Policy direction and expectations are clear: shifting away from competition towards more collaborative ways of working (7). Examples include recent National Institute for Health and Care Excellence (NICE) standards to facilitate effective partnership working (8), 'flu vaccine principles (9), and enhanced service amendments (10) for GP referrals to CP. However, implementing these changes has created unanticipated and unpredictable consequences. For instance, the more clinical activity is distributed across providers, healthcare disciplines, and settings, the greater the risk for silo working, fragmentation of accountability and patient care (11). To enable effective collaborative and integrated working, there remains a need to

For peer review only

develop an in-depth understanding about how these providers work together; what influences interactions; and how these impact patient trust, experience, equity, and outcomes.

UK reports in the last decade consistently position CP as a solution for an over-burdened GP and a means to improve access for patients from socioeconomically deprived communities, providing 'health on the high street' (12-14) including Directed Enhanced Services (15). This has been part of the rationale behind the Long-Term Plan's goal of reorganising primary care so that CP is included in wider networks such as Primary Care Networks (PCNs) and ICSs (1). However, implementation of collaborative working amongst independent contractors to the NHS, such as GP and CP, can be complex and problematic.

Professional bodies, including the Royal Pharmaceutical Society (16), have published recommendations to help CP integrate into primary care (17). However, these are not specific to ICSs and PCNs, and predominantly focus on tighter technology integration to facilitate communication between GP and CP. Recent guidance (9) includes expectations for integrated services, referral pathways, and joint strategic needs assessments to address local health inequalities, but does not describe how these should be achieved.

Policy changes have the potential to influence how patients' access, use, and experience primary care; form and sustain relationships with healthcare professionals; and navigate healthcare expertise within and between clinical encounters. This has implications for the nature of work done by professional groups including how this work is achieved, the use of technology to support working, how accountability (clinical and financial) is determined, the training and capacity building required; and importantly, how healthcare professionals work with each other to support effective, safe, and equitable patient care.

Previous reviews have demonstrated that CP could deliver effective patient care in several areas, but implementation depends on successful collaboration and integration with other services (6, 18). Existing research into collaborative working in these settings found

For peer review only

that imbalances of authority, limited understanding of others' roles and responsibilities, lack of time and financial remuneration models, and professional boundary friction when delivering patient care, can impact on the quality of care provided (6, 19, 20). Collaboration and integrated working may bring new roles and ways of working for healthcare professionals in each organisation. These changes may also, however, contribute to and exacerbate ambiguity about professional roles and boundaries, potentially undermining the collaborative and integrated working between GP and CP, with resulting confusion for patients (21). There is, therefore, a potential paradox at play, in which efforts to create greater role clarity, professional boundaries, and distribution of work tasks in the service of integrating care contribute to poorly integrated patient care. Extant literature is not, however, able to explain when (in what contexts), why, and how the identified factors affect working relationships between GP and CP. Whilst current evidence indicates that encouraging GP and CP to work together is important, it is less clear how these working arrangements can be made to work well within ICS settings for both healthcare professionals and patients from diverse cultural and socioeconomic backgrounds.

For peer review only

METHODS

Review aim, questions, and objectives

Aim

This review aims to understand how, when, and why working arrangements between GP and CP can provide the conditions necessary for optimal communication, decision-making, and collaborative and integrated working.

Research Objectives

1. Develop a programme theory through an evidence synthesis of how GP and CP can optimise communication, decision-making, and collaborative and integrated working to support effective and equitable patient care.
2. Embed and respond to stakeholder and PPIE perspectives throughout the design, analysis, and report stages of the project, thus maximising the relevance and utility of review findings.
3. Make recommendations for practice and policy based on the refined programme theory.

Research Questions:

Within the existing literature, what can we learn that will help GP and CP to work together in a collaborative and integrated way to support effective and equitable healthcare outcomes? Specifically, the review will be guided by the following questions:

1. What are the mechanisms which support GP and CP to work in an integrated and collaborative way?

2. What are the important contexts which influence whether different mechanisms produce intended and unintended outcomes in GP and CP working relationships?

3. What are the interventional strategies that are likely to lead to intended and unintended outcomes within GP and CP working relationships?

Approach

A realist review is an interpretive and theory-driven approach to synthesising evidence from grey literature (e.g., policy documentation), qualitative, quantitative, and mixed-methods research (22). It enables the use of a range of data types to make sense of and address the context-sensitive outcomes arising from interactions between GP and CP. The

For peer review only

realist approach begins by developing an initial programme theory that aims to explain how collaborative and integrated working between GP and CP may (or may not) work, and includes theorising the anticipated interactions between contexts, mechanisms, and outcomes (23). This initial programme theory is then tested (confirmed, refuted, or refined) against empirical evidence throughout the review. The final phase of a realist review involves the synthesis of evidence, the formulation of context-mechanism-outcome configurations (CMOCs), and a refined programme theory which explains whether, why, how, and to what extent GP and CP collaborative and integrated working practices may (or may not) support effective and equitable healthcare delivery for patients (23). A realist review has the potential to produce meaningful, transferable findings across different structures and contexts within which GP and CP operate.

Patient and Public Involvement and Engagement

We will consult with stakeholders with a wide range of relevant expertise comprising: four PPIE co-applicants (including family and carer perspectives); front-line healthcare clinicians; workforce and training experts; and a multi-disciplinary team of researchers. The project team (including PPIE co-applicants) will meet every two months, working collaboratively alongside experiential stakeholders. There will be three stakeholder meetings (lasting approximately 3-hours), including representation from small, medium, and large pharmacy and medical organisations. Throughout the project, detailed notes will be made during and after each meeting, and the outcomes of our engagement with PPIE co-applicants and stakeholders will be recorded using an impact log (24). Collectively, our PPIE co-applicants and stakeholder groups will contribute to the development and refinement of the programme theory, analysis, interpretation, dissemination activities (e.g., co-presenting and co-publishing papers, posters, presentations, and reports), and help to ensure our research informs future practice and policy in this important area of inquiry. This realist review

For peer review only

protocol is registered in PROSPERO (reference number: CRD42022314280) and will be carried out in accordance with the quality and publication standards (25, 26).

Step 1: Finding Existing Theories

We will identify theories that explain how collaborative and integrated working between GP and CP is expected to 'work' to generate effective and equitable healthcare outcomes (25). To do this, we will consult with key content experts in our stakeholder group and informally scope existing literature identified via citation tracking and snowballing (27), which will also include identifying any formal theories that may be of relevance (28). We will use relevant theories and data we find in this step to develop our initial programme theory, which will be further tested and scrutinised in this review. A draft of our initial programme theory is provided in Figure 1.

Figure 1 Initial programme theory – used at the beginning of realist review projects to map initial explanatory theories.

****Insert Figure 1 Around Here****

Step 2: Searching for Evidence

We will conduct systematic searches to identify a relevant 'body of literature' with which to develop and refine the programme theory from step 1. The main search strategy was designed, piloted, and conducted by CD in collaboration with ECO and the project team. Our searches combined free text and subject heading (e.g., MeSH) terms for GP and CP with a range of terms describing collaborative and integrated working arrangements. The full details of the search developed for MEDLINE are available in Supplementary File 1. This search strategy was translated for use in the following databases: MEDLINE (Ovid); Embase

For peer review only

(Ovid); CINAHL (Ebsco); PsycINFO (Ovid); HMIC (Ovid); International Bibliography of the Social Sciences (ProQuest); Sociology Collection (ProQuest); the Web of Science (SSIE, SSCI, ESCI, and CPCI indexes); and the King's Fund Library database. This search identified 2555 potentially relevant documents, of which 1133 were duplicates. A total of 1422 unique references were identified for screening.

At later stages of this review, we may undertake additional searching, including searches of any other relevant databases identified by CD, 'cited by' article searches, and searches of the citations contained in the reference lists of relevant documents. Additional grey literature e.g., documents produced by the Department of Health, local Clinical Commissioning Groups (or successor organisations), and pharmacists' professional groups will also be identified via searches of relevant websites. Relevant evidence including opinion and commentary will be used to inform programme theory development, along with published and unpublished evaluations of existing integrated services and collaborations which are likely to provide more detailed local contextual information.

Based on our preliminary searches, our initial inclusion criteria will include GP and CP; UK (the initial focus will be on the UK and countries with a universal healthcare system but we may draw on data from other healthcare systems); Date 2000 onwards (in order to capture literature prior to the first integrated and collaborative initiative called Medicines Use Review in 2003); and a focus on an element of the 'working relationship' between GP and CP (to include terms such as integrated and collaborative working, but not exclusively). We expect to exclude papers focused entirely on clinical pharmacists working within GP settings due to differences in roles, responsibilities, and the professional framework of community and clinical pharmacists.

Step 3: Article Selection

For peer review only

This is a three-stage process: screening against title/abstract; then by full text; and finally full text documents will be selected based on their relevance (whether they contain data to contribute to theory building and/or testing) and rigour (whether the methods used to generate the relevant data are credible and trustworthy – for instance, depending on the type of document, appropriate quality standards will be used in addition to regular research team discussions) (22, 25) To ensure consistency, a random 10% sample of decisions will be independently checked at each stage by CD and/or NF. Any discrepancies or disagreements will be discussed with the research team and documented.

Step 4: Data Extraction

Data extraction and organisation will be undertaken by ECO. Discrepancies or disagreements will be discussed between the research team in detail and documented. The included full texts will be uploaded into qualitative data analysis software (EPPI-Reviewer Web) for coding. These will be both coded deductively (codes created in advance of data extraction and analysis, as informed by the initial programme theory), inductively (codes created to categorise data reported in included studies), and retroductively (codes created based on an interpretation of data to infer what the hidden causal forces might be for outcomes). Each new element of relevant data will be used to refine the programme theory, and as it is refined, included studies will be re-scrutinised to search for relevant data that may have been missed initially. A random sample of 10% extracted data and coding will be independently checked by CD or NF for quality control.

Step 5: Synthesising the Evidence and Drawing Conclusions

Data analysis will use a realist logic of analysis to make sense of the initial programme theory. ECO will undertake this step with support from the research team, PPIE,

For peer review only

and stakeholders. We will use interpretive cross-case comparison to understand and explain how and why observed outcomes have occurred, for example, by comparing literature in which GP and CP have successfully worked collaboratively against those which have reported the interface as unsuccessful or detrimental, to understand how context has influenced reported findings.

We will use an established analysis and synthesis process (28). In brief, to operationalise the realist logic of analysis, we will ask the following questions:

- Interpretation of meaning: do the documents provide data that may be interpreted as functioning as context, mechanism, or outcome?
- Interpretations and judgements about CMOCs: what is the CMOC for the data that has been interpreted as functioning as context, mechanism, or outcome?
- Interpretations and judgements about programme theory: how does this CMOC relate to the initial programme theory?

Ethics and Dissemination

Dissemination

Our PPIE contributors and stakeholders will help us to decide on the content, storyboarding, and format (e.g., websites, leaflets, videos, social media). In addition to the final report, we will produce outputs directed at diverse audiences:

1. **Academic outputs**: e.g., protocol publication. Findings (to be submitted to a high-impact, open-access, and peer-reviewed journal), as well as tailored papers to different disciplinary journals.
2. **Audience-specific practitioner** ‘how to’ publications which outline practice advice on how to optimise GP and CP collaborative and integrated working for patient benefit.

For peer review only

3. **User-friendly summaries** of the review findings tailored to the needs of different audiences including the public and service users.

Our dissemination strategy will build upon a participatory approach, embedding PPIE and stakeholder involvement throughout the development of this research and project timeline, including opportunities for co-authorship and co-presenting. On-going engagement with key stakeholders will maximise opportunities to utilise our established networks, communication channels, and links to policymakers and providers. Our approach will be integrative, valuing the different forms of knowledge needed to produce findings capable of informing complex decision-making (29).

Ethics

Ethical approval is not required for this review as only secondary data sources will be used.

DISCUSSION

Importance of the Research

This review will provide insights and recommendations to maximise GP and CP collaboration and integration. Importantly, it will help to identify where critical knowledge 'gaps' exist and propose ways to close these gaps, providing direction for future theoretical research and practice. The findings and refined programme theories will ensure patients' health and healthcare experience is central to GP and CP working relationships and processes. These working relationships and arrangements impact on patient experience, patient safety and medication errors, access, care, and formal referral, alongside professional capacity, training, and workload. The review findings are likely to have broader relevance to

other primary care interfaces and the future productive shaping of collaborative and integrated working.

Authors' contributions

The realist review was conceptualised by SP. ECO wrote the first draft of this protocol and it was reviewed and revised by SP, CW, CD, DS, FH, GW, JHH, KRM, MT, MO, NF, RA, ZC. CD designed, piloted, and conducted the search strategy in collaboration with ECO and the project team. All authors have read and approved the final manuscript.

Funding Acknowledgement

This project is funded by the National Institute for Health Research (NIHR) School for Primary Care Research (project reference 567988). The views expressed are those of the authors and not necessarily those of the NIHR or the Department of Health and Social Care.

Competing interests

The authors have no conflicts of interests to declare.

For peer review only

References

1. NHS E. The NHS Long Term Plan 2019 [Available from: https://www.longtermplan.nhs.uk/wp-content/uploads/2019/08/nhs-long-term-plan-version-1.2.pdf.

2. Health Do. A vision for pharmacy in the new NHS 2003 [Available from: http://data.parliament.uk/DepositedPapers/Files/DEP2009-0485/DEP2009-0485.pdf.

3. Mossialos E, Courtin E, Naci H, Benrimoj S, Bouvy M, Farris K, et al. From "retailers" to health care providers: Transforming the role of community pharmacists in chronic disease management. Health Policy. 2015;119(5):628-39.

4. Morley L, Cashell A. Collaboration in Health Care. J Med Imaging Radiat Sci. 2017;48(2):207-16.

5. Boon HS, Mior, S. A, Barnsley, J, Ashbury, F. D, Haig, R. The difference between integration and collaboration in patient care: results from key informant interviews working in multiprofessional health care teams. J Manipulative Physiol Ther. 2009;32(9):715-22.

6. Bollen A, Harrison R, Aslani P, van Haastregt JCM. Factors influencing interprofessional collaboration between community pharmacists and general practitioners-A systematic review. Health Soc Care Community. 2019;27(4):e189-e212.

7. NHS E. We are the NHS: people plan 2020-21—action for us all. 2020 [Available from: https://www.england.nhs.uk/publication/we-are-the-nhs-people-plan-for-2020-21-action-for-us-all/.

8. (NICE) NIfHaCE. Community pharmacies: promoting health and wellbeing 2020 [Available from: https://www.nice.org.uk/guidance/qs196/resources/community-pharmacies-promoting-health-and-wellbeing-pdf-75545785198789.

9. Practitioners RPSaRCoG. Influenza vaccination: Principles for collaboration across Great Britain 2020 [Available from: https://www.rpharms.com/Portals/0/RPS%20document%20library/Open%20access/Flu%20vaccinations/Flu%20Vaccination%20Joint%20Statement.pdf.

10. ACT TNHS. The Pharmaceutical Services (Advanced and Enhanced Services and Emergency Declaration) (England) (Amendment) (No. 2) Directions 2020
2006 [Available from: https://www.nhsbsa.nhs.uk/sites/default/files/2020-08/Pharmaceutical%20Services%20%28Adv%20Enh%20%20Emerg%20Declar%29%20%28Eng%29%20%28Flu%20vacc%20Hep%20C%20etc%29%20Dir%2028082020.pdf.

11. Sevin C, Moore G, Shepherd J, Jacobs T, Hupke C. Transforming care teams to provide the best possible patient-centered, collaborative care. J Ambul Care Manage. 2009;32(1):24-31.

12. Alliance N. Supporting the Development of Community Pharmacy Practice within Primary Care 2016 [Available from: https://nhsalliance.org/wp-content/uploads/2018/11/Supporting-the-Development-of-Community-Pharmacy-Practice-within-Primary-Care-NEW-LOGO.pdf.

13. Smith J, Picton, C., Dayan, M NOW OR NEVER: shaping pharmacy for the future. 2013

14. Todd A, Copeland A, Husband A, Kasim A, Bambra C. The positive pharmacy care law: an area-level analysis of the relationship between community pharmacy distribution, urbanity and social deprivation in England. BMJ Open. 2014;4(8):e005764.

15. Committee DPSN. A summary of the Network Contract Directed Enhanced Service (DES) 2019/20 2019 [Available from: https://psnc.org.uk/wp-content/uploads/2019/04/PSNC-Briefing-018.19-A-summary-of-the-Network-Contract-Directed-Enhanced-Service-DES-2019.20.pdf.

For peer review only

16. Society. RP. The future of pharmacy in a sustainable NHS: Key principles for transformation and growth 2022 [Available from: https://www.rpharms.com/recognition/all-our-campaigns/policy-a-z/future-of-pharmacy.
17. Body PRS. Community Pharmacy Standard Final Report 2022.
18. Milosavljevic A, Aspden T, Harrison J. Community pharmacist-led interventions and their impact on patients' medication adherence and other health outcomes: a systematic review. Int J Pharm Pract. 2018;26(5):387-97.
19. Hossain LN, Fernandez-Llimos F, Luckett T, Moullin JC, Durks D, Franco-Trigo L, et al. Qualitative meta-synthesis of barriers and facilitators that influence the implementation of community pharmacy services: perspectives of patients, nurses and general medical practitioners. BMJ Open. 2017;7(9):e015471.
20. Hindi AMK, Schafheutle EI, Jacobs S. Community pharmacy integration within the primary care pathway for people with long-term conditions: a focus group study of patients', pharmacists' and GPs' experiences and expectations. BMC Fam Pract. 2019;20(1):26.
21. Karampatakis GD, Patel N, Stretch G, Ryan K. Community pharmacy teams' experiences of general practice-based pharmacists: an exploratory qualitative study. BMC Health Serv Res. 2020;20(1):431.
22. Pawson R. Evidence-Based Policy: A Realist Perspective London: SAGE Publications.; 2006.
23. Pawson R, & Tilley, N Realistic Evaluation SAGE Publications; 1997.
24. Kok M. Guidance Document: Evaluating public involvement in research 2018 [Available from: http://www.phwe.org.uk/wp-content/uploads/Guidance-on-evaluating-Public-Involvement-in-research.pdf.
25. Wong G, Greenhalgh T, Westhorp G, Buckingham J, Pawson R. RAMESES publication standards: realist syntheses. BMC Med. 2013;11:21.
26. Wong G, Greenhalgh T, Westhorp G, Pawson R. Development of methodological guidance, publication standards and training materials for realist and meta-narrative reviews: the RAMESES (Realist And Meta-narrative Evidence Syntheses - Evolving Standards) project. Health Services and Delivery Research. Southampton (UK)2014.
27. Greenhalgh T, Peacock R. Effectiveness and efficiency of search methods in systematic reviews of complex evidence: audit of primary sources. BMJ. 2005;331(7524):1064-5.
28. Booth A, Harris J, Croot E, Springett J, Campbell F, Wilkins E. Towards a methodology for cluster searching to provide conceptual and contextual "richness" for systematic reviews of complex interventions: case study (CLUSTER). BMC Med Res Methodol. 2013;13:118.
29. Bowen SG, I Integrated knowledge translation. In: Straus S, & Tetroe, J editor. Knowledge translation in health care: Moving from evidence to practice. Oxford: Wiley Blackwell 2013 p. 14-23.

For peer review only

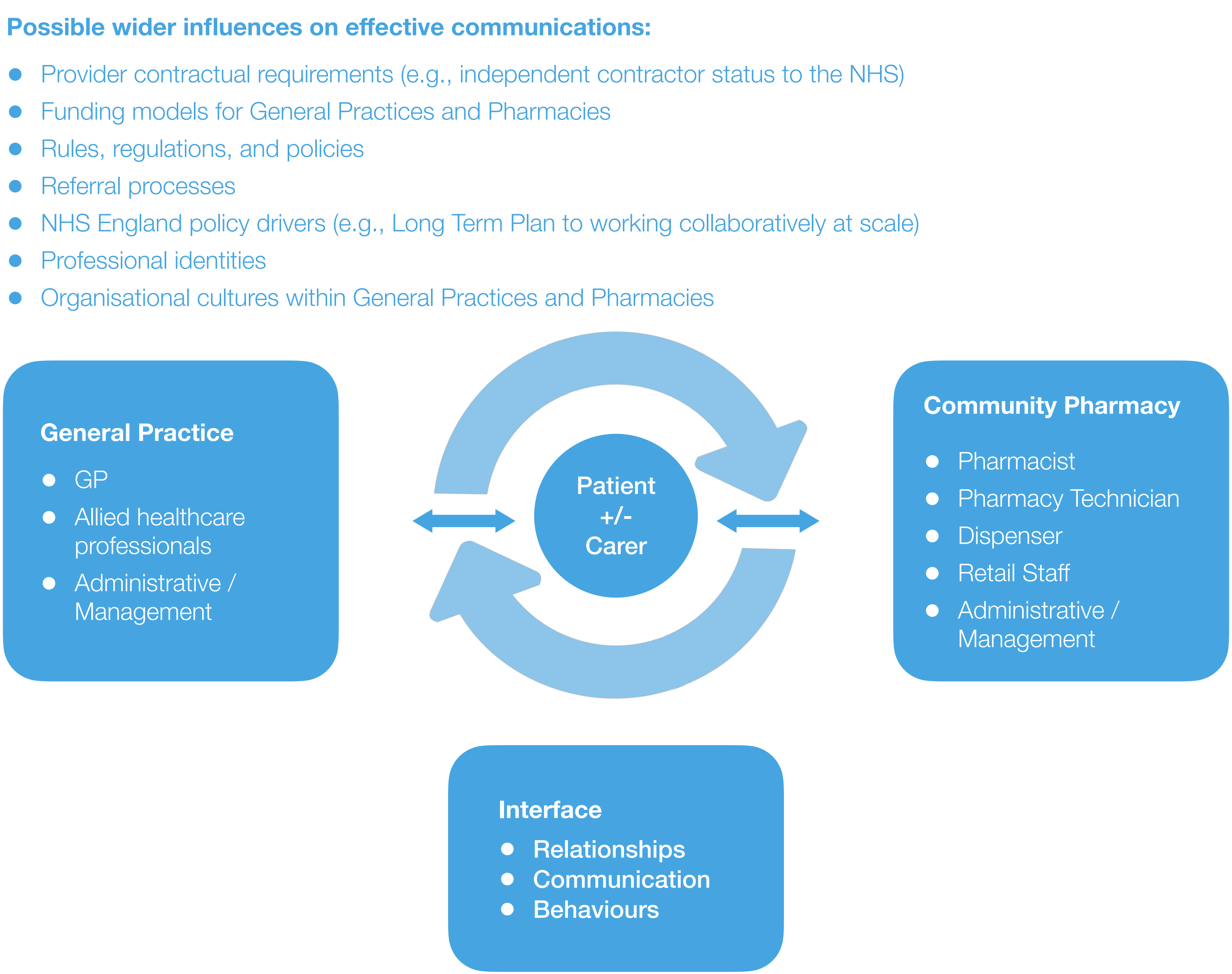
Possible wider influences on effective communications:
Provider contractual requirements (e.g., independent contractor status to the NHS)
Funding models for General Practices and Pharmacies
Rules, regulations, and policies
Referral processes
NHS England policy drivers (e.g., Long Term Plan to working collaboratively at scale)
Professional identities
Organisational cultures within General Practices and Pharmacies
General Practice
GP
Allied healthcare professionals
Administrative / Management
Patient +/- Carer
Community Pharmacy
Pharmacist
Pharmacy Technician
Dispenser
Retail Staff
Administrative / Management
Interface
Relationships
Communication
Behaviours

Supplementary File 1

Details of the search developed for MEDLINE:

**Data Source**	Database	MEDLINE	
	Details	Medline (Ovid MEDLINE® Epub Ahead of Print, In-Process & Other Non-Indexed Citations, Ovid MEDLINE® Daily and Ovid MEDLINE®) 1946 to present	
	Host	Ovid	
	Date searched	18/04/2022	
	Database Update	Daily update	
**Limiters**	English only?	Yes	
	Time period searched	2000 to current	
	Publications types	No limit	
	Other		
**Results**	Items found	609	
	Internal duplicates (within one database)	0	
	External duplicates (between databases)	0	
	New	609	
In Ovid, save your search. Go to **Saved Searches** and click on the eye icon of your SR saved search to **View** your search. Copy and paste your search into **Notepad;** put tabs between the line number and text. Copy and paste into cell **B15**.	**Paste text of search strategy below**		
	**CompanionTest Version E MEDLINE**		
	Medline (Ovid MEDLINE® Epub Ahead of Print, In-Process & Other Non-Indexed Citations, Ovid MEDLINE® Daily and Ovid MEDLINE®) 1946 to present		
	1	((communit* or local) adj2 pharmac*).ti,ab,kw.	10064
	2	Community Pharmacy Services/	5409
	3	1 or 2	11999
	4	(general practi* or family practi* or family physician* or primary health* or primary care).ti,ab,kw.	249834
	5	exp *Primary Health Care/	106846
	6	exp *General Practice/	50899
	7	*general practitioners/ or *physicians, family/ or *physicians, primary care/	22059
	8	*Ambulatory Care/	20715
	9	or/4-8	348227
	10	((integrat* or collab* or cooperat* or co-operat* or coordinat* or co-ordinat* or interprofessional* or inter-professional* or multidisciplin* or multi-	158988
	11	((joint or joined-up or joined up or relations*) adj1 work*).ti,ab,kw.	4322
	12	((organi?ation* or delivery or care) adj1 model*).ti,ab,kw.	14362
	13	(infrastructure* or interface*).ti,ab,kw.	267499
	14	*Attitude of Health Personnel/	65859
	15	*Cooperative Behavior/	18726
	16	exp *Interprofessional Relations/	29750
	17	exp *Professional Role/	46346
	18	exp *"Delivery of Health Care, Integrated"/	10524
	19	*models, organizational/	6488
	20	or/10-19	583426
	21	9 and 20	43231
	22	Primary Health Care/og [Organization & Administration]	15886
	23	21 or 22	54656
	24	3 and 23	669
	25	limit 24 to (english language and yr="2000 -Current")	609

For peer review only
